# Upregulation of CD244 promotes CD8^+^ T cell exhaustion in patients with alveolar echinococcosis and a murine model

**DOI:** 10.1186/s13071-024-06573-2

**Published:** 2024-11-23

**Authors:** Maolin Wang, Bingqing Deng, Tiemin Jiang, Adilai Duolikun, Yinshi Li, Abidan ainiwaer, Xuejiao Kang, Xuran Zheng, Zibigu Rousu, Qian Yu, Jing Li, Hui Wang, Chuanshan Zhang, Tuerganaili Aji, Yingmei Shao

**Affiliations:** 1 Department of Hepatobiliary and Echinococcosis Surgery, Digestive and Vascular Surgery Center, First Affiliated Hospital of Xinjiang Medical University, Urumqi, 830054 China; 2 State Key Laboratory of Pathogenesis, Prevention and Treatment of High Incidence Diseases in Central Asia, Xinjiang Medical University, Clinical Medicine Institute, The First Affiliated Hospital of Xinjiang Medical University, Urumqi, 830054 China; 3 Clinical Medical Research Center of Echinococcosis and Hepatobiliary Disease of Xinjiang Uygur Autonomous Region, Urumqi, 830054 China; 4 Basic Medical College, Xinjiang Medical University, Urumqi, 830017 China

**Keywords:** Alveolar echinococcosis, CD8, Immune exhaustion, CD244

## Abstract

**Background:**

In patients with alveolar echinococcosis (AE), CD8^+^ T cells undergo functional exhaustion, which accelerates the malignant progression of AE. However, the role of inhibitory receptor CD244 in mediating CD8^+^ T cell exhaustion remains elusive.

**Methods:**

CD244 expression on exhausted CD8^+^ T cells in the close liver tissue (CLT) of AE patients was analyzed using single-cell RNA sequencing data. Immunohistochemistry and immunofluorescence were employed to detect CD244 expression. Flow cytometry was used to assess the impact of CD244 on differentiation and effector function of CD8^+^ T cells in patients with AE, in vitro and in vivo models. Reactive oxygen species (ROS) and oxygen consumption rate (OCR) were measured to evaluate the influence of CD244 on mitochondrial function of CD8^+^ T cells.

**Results:**

CD244^+^CD8^+^ T cells in the CLT of AE patients exhibit a more terminal differentiation phenotype, with reduced secretion of IFN-γ and TNF-α. In vitro studies revealed that CD8^+^ T cells from CD244-deficient mice produced higher levels of IFN-γ, TNF-α and Granzyme B. In vivo studies revealed that CD244 deficiency enhanced the secretion capacity of IFN-γ and TNF-α by CD8^+^ T cells, inhibiting the growth of metacestodes. Moreover, CD244 deficiency leads to a decrease in ROS levels in liver CD8^+^ T cells, while significantly increasing their adenosine triphosphate (ATP)-linked oxygen consumption rate.

**Conclusions:**

CD244 facilitates AE disease progression by mediating immune exhaustion in CD8^+^ T cells.

**Graphical abstract:**

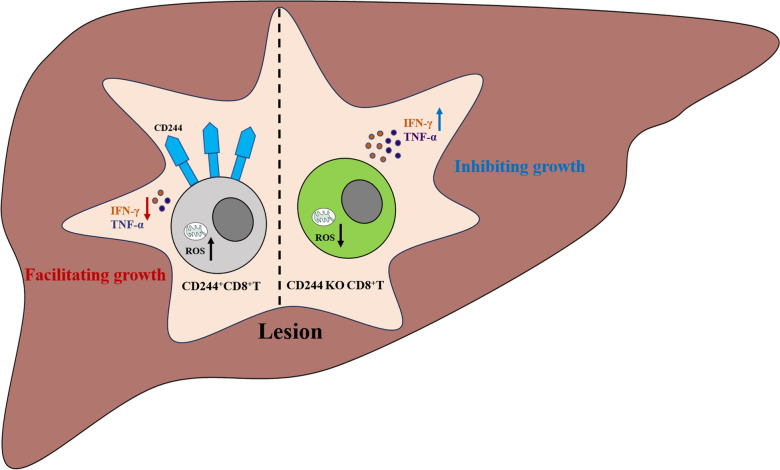

**Supplementary Information:**

The online version contains supplementary material available at 10.1186/s13071-024-06573-2.

## Background

Alveolar echinococcosis (AE) is a fatal zoonosis caused by the metacestode larvae of *Echinococcus multilocularis* (*E.* *multilocularis*) [1]. Owing to the high mortality associated with AE [2], the World Health Organization (WHO) has recognized it as one of the 17 neglected diseases designated for control or elimination by 2050 [3]. Predominantly, over 97% of AE cases are hepatic [4], characterized by budding growth in the germinal layer of metacestodes, leading to hepatic tissue destruction and tumor-like lesion formation. The optimal treatment for hepatic AE combines radical hepatectomy with oral albendazole [5]. However, the insidious onset and slow progression of AE often cause patients to miss the optimal timing for surgery [6]. In such scenarios, oral albendazole serves as an alternative therapeutic option [7], but its efficacy is limited, highlighting the urgent need for further research into the pathogenic mechanisms of hepatic AE and the development of more effective therapeutic strategies.

The persistent presence of *E. multilocularis* leads to the exhaustion of CD8^+^ T cells, with potential mechanisms that include: (i) metabolic abnormalities such as oxidative stress leading to T cell functional exhaustion [8]; (ii) activation and proliferation of Tregs promoting CD8^+^ T cell functional exhaustion [9]; (iii) increased expression of inhibitory receptors (IRs) suppressing the activation and proliferation of CD8^+^ T cells [10]; (iv) elevated secretion of inhibitory cytokines such as interleukin-10 (IL-10) and transforming growth factor beta (TGF-β) in the immune microenvironment can inhibit the function of CD8^+^ T cells [11]. Among these mechanisms, the role of IRs in the functional exhaustion of CD8^+^ T cells during parasitic infections has garnered increasing attention in recent years.

Recent studies have demonstrated that the upregulation of immune checkpoints, such as programmed cell death 1 (PD-1) and T cell immunoreceptor with immunoglobulin and immunoreceptor tyrosine-based inhibitory motif domain (TIGIT), is instrumental in driving CD8^+^ T cell exhaustion during *E. multilocularis* infection [12, 13]. CD244 (2B4), a distinct and newly recognized immune checkpoint, is increasingly the focus of research interest. As a crucial member of the signaling lymphocyte activation molecule (SLAM) family, CD244 is primarily expressed on the surface of NK cells, γδT cells, basophils, monocytes, some CD8^+^ αβ T cells, dendritic cells and myeloid-derived suppressor cells (MDSCs) [14–16]. In fact, the complex immunoregulatory function of CD244 allows it to exert dual effects of promotion and inhibition on different immune cells, depending on the expression level of CD244 and its interaction with downstream signaling proteins [SLAM associated protein (SAP) or Ewing sarcoma-activated transcript 2 (EAT2)] [17–19].

In the study of chronic viral infections, it was found that low to moderate expression of CD244 can promote the effector function of lymphocytic choriomeningitis virus (LCMV)-specific CD8^+^ T cells, while high expression of CD244 can inhibit the effector function of its specific CD8^+^ T cells [20]. Furthermore, research suggests that the lower SAP/CD244 expression ratio in peripheral blood CD8^+^ T cells of patients with acute lymphocytic leukemia indicates an inhibitory role of CD244 [21]. Additionally, the results of intraperitoneal tumor clearance experiments indicate that EAT2-deficient mice boast a stronger ability to clear myeloma cells compared to wild-type (WT) mice, suggesting that the binding of CD244 with EAT-2 plays an immunosuppressive role [17]. Our previous findings indicated that the expression level of CD244 is elevated in exhausted CD8^+^ T cells during chronic *E. multilocularis* infection [22]. However, the detailed role of CD244 in mediating CD8^+^ T cell exhaustion in *E. multilocularis* infection remains unclear.

In our study, we observed increased expression of CD244 on CD8^+^ T cells from close liver tissues (CLT) in patients with AE. These CD244^+^CD8^+^ T cells also showed reduced secretion of the inflammatory cytokines interferon-gamma (IFN-γ) and tumor necrosis factor-alpha (TNF-α). Furthermore, we found that knocking out CD244 somewhat inhibited the growth of metacestodes in the late stages. Furthermore, CD244 deficiency was shown to inhibit the terminal differentiation process and restore effector function of CD8^+^ T cells. Our study preliminarily elucidates that CD244 mediates CD8^+^ T cell immune exhaustion in persistent *E. multilocularis* infection by driving the terminal effector phenotype.

## Methods

### Bioinformatics analysis

We conducted bioinformatics analysis using shared single-cell sequencing data from AE patients (https://ngdc.cncb.ac.cn/gsa-human/browse/HRA000553). Data processing and analysis were performed using R software (R version 4.3.0).

### Sample preparation

Consistent with protocols from our previous research [12], we collected one specimen adjacent to the parasitic lesion, referred to as close liver tissue (CLT), approximately 0.5 cm from the lesion. Additionally, a specimen from the macroscopically normal liver tissue, referred to as distant liver tissue (DLT), was taken at least 2 cm away from the lesion. Comprehensive details of the data collected from AE patients are presented in Table S1. The number of liver tissue samples, types of measurements and the objectives of the comparisons are outlined in Table S2.

### Mouse, parasite, *E. multilocularis* infection model

Female C57BL/6 WT mice, aged 8 weeks, were sourced from Beijing Vital River Experimental Animal Technology Co., Ltd. C57BL/6 CD244-knockout (KO) mice were obtained from GemPharmatech Co., Ltd., in Nanjing, China. Additionally, C57BL/6 CD8-KO mice, along with CD45.1 WT mice, were generously provided by Dr. Zhexiong Lian from the Guangdong Academy of Medical Sciences, China. Breeding between female CD45.2 WT mice and male CD45.1 mice resulted in the generation of CD45.1/2 WT mice. All mice were housed in the specific pathogen-free facility at the Animal Experimental Center of Xinjiang Medical University.

*Echinococcus multilocularis* protoscoleces (PSCs) were meticulously prepared and harvested from the abdominal cavities of infected Mongolian gerbils in a controlled, sterile environment. The mice were inoculated intravenously through the portal vein with a biologically active suspension of PSCs in saline solution, according to an established protocol [22]. Conversely, the control group of mice received injections of saline solution.

### Immunohistochemistry

Liver sections were deparaffinized in xylene, rehydrated through a graded ethanol series and finally rinsed in deionized water, each step lasting for 2 min. Antigen retrieval was carried out using citric acid buffer for 15 min. Sections were subsequently blocked with PBS and 10% donkey serum at room temperature for 1 h. Primary antibodies, anti-human CD244 (R&D Systems, 1:500), were applied at appropriate dilutions and incubated overnight at 4 °C. The next day, sections were washed and incubated with secondary antibodies (donkey anti-goat F(ab’)2-HRP, 1:500) for 2 h at room temperature. DAB staining was developed following Abcam’s kit instructions. The proportion of positively stained regions was quantified using CellSens Dimension software (Olympus, Japan) at 100× or 400× magnification. For each section, 3–5 fields of view were captured for measurement, and the average was calculated for subsequent statistical analysis.

### Isolation of mononuclear cells from mouse liver and spleen

The livers of the mice were homogenized using a 200-mesh steel sieve. Following low-speed centrifugation, the cell pellet was retained and resuspended in 40% lymphocyte separation solution (GE Healthcare, Little Chalfont, UK), followed by gradient centrifugation at 25 °C for 30 min. The mouse spleens were crushed and ground using the frosted surfaces of two microscope slides. Subsequently, the spleen cells were resuspended in phosphate buffer saline (PBS) and filtered through a 200-mesh nylon filter. Red blood cell lysis buffer was employed to deplete red blood cells. After terminating the lysis, single-cell suspension was centrifuged, and the pellet was resuspended in PBS containing 0.2% bovine serum albumin (BSA) to obtain mononuclear cells. After being stained with trypan blue, cell counting was performed using a hemocytometer.

### Flow cytometry (FCM)

Single-cell suspensions from the liver and spleen tissues of mice infected with *E. multilocularis* were treated with a PBS buffer containing 0.2% BSA and 0.1% sodium azide. These suspensions were then incubated with Fc Block and monoclonal antibodies against CD16:CD32 for 30 min at 4 °C to prevent nonspecific antibody binding. Following Fc receptor blockade, the cells were stained for surface markers, intracellular cytokines and nuclear components following established methodologies [23]. The stained cells were analyzed by flow cytometry, and the data were meticulously examined. Details on the antibodies used are listed in Table S3. Flow cytometry gating strategies are presented in Fig. S6.

### *Echinococcus multilocularis* antigen preparation and in vitro stimulation

*Echinococcus multilocularis* PSCs (Emp) antigens were prepared using a standard method [12]. In a sterile biosafety cabinet, liver lymphocytes from WT and CD244-KO mice were isolated and cultured in a 96-well plate, with 250 μl of complete culture medium added to each well. The medium contained Emp, anti-CD3 and anti-CD28 at working concentrations of 1 μg/ml, 0.8 μg/ml and 1.6 μg/ml, respectively. The cells were stimulated at 37 °C for 2 days, and brefeldin A (1000× dilution, BD Biosciences) was added during the last 12 h to inhibit cytokine secretion. After stimulation, the cells were harvested for determining the intracellular cytokine production (IFN-γ, TNF-α and IL-10) of CD8^+^ T cells, CD4^+^ T cells and NK cells via flow cytometry.

### Detection of ROS levels in mouse lymphocytes

Add 1 × 10^6^ hepatic or cells to a 1.5 ml centrifuge tube; centrifuge at 6000 rpm for 2 min and discard the supernatant. Prepare the staining solution by mixing RPMI 1640 culture medium with MitoSOX™ Red mitochondrial superoxide indicator (Invitrogen by Thermo Fisher Scientific) at a ratio of 1000:1, and add 100 μl of this staining solution to the centrifuge tube. Mix thoroughly, and then open the lid and incubate it in a 37 °C CO_2_ incubator for 30 min. After staining is complete, proceed with the steps for blocking and antibody labeling. After resuspension and transfer to a new tube, use a flow cytometer for detection.

### Seahorse metabolic analysis

Seahorse cell culture plates were coated with Cell-Tak (Corning, 22.4 μg/ml) as per the manufacturer’s instructions. Cell counting was performed using a hemocytometer, and 5 × 10^5^ viable CD8^+^T cells were plated per well as per the manufacturer’s instructions. Seahorse media used consisted of glucose (1 M), glutamine (200 mM) and sodium pyruvate (100 mM). The oxygen consumption rate (OCR) was measured before and following injection with oligomycin (1 μM), carbonyl cyanide-4-(trifluoromethoxy) phenylhydrazone (FCCP, 2 μM) and antimycin-A/rotenone (0.5 μM) using the Seahorse XFe24 analyzer.

### Adoptive transfer of CD8^+^ T cells

Lymphocytes were isolated from the spleen tissues of CD244-KO (CD45.2) and WT (CD45.1/2) mice followed by the purification of CD8^+^ T cells using Magnetic-Activated Cell Sorting. A total of 1 × 10^6^ cells from each genotype were intravenously transferred into two distinct groups of CD8-KO recipient mice, each with a 30-day *E. multilocularis* infection. One month after the transfer, the recipient mice were killed to evaluate the CD8^+^ T-cell response in both liver and spleen tissues.

### Statistical analysis

Statistical analysis was performed using GraphPad Prism 8.3.0 software (GraphPad Software, San Diego, California), and results were presented as mean ± standard deviation. Student’s t-test (parametric) or Mann-Whitney *U* test (nonparametric) was used to compare two separate groups. The one-way ANOVA or Kruskal-Wallis test was used when there were more than two groups. Paired independent samples Student’s t-tests were utilized to analyze data within groups. For all experimental outcomes, a *P* value < 0.05 was considered statistically significant.

## Results

### Upregulation of CD244 contributes to CD8^+^ T cell exhaustion in hepatic AE patients

**Fig. 1:**
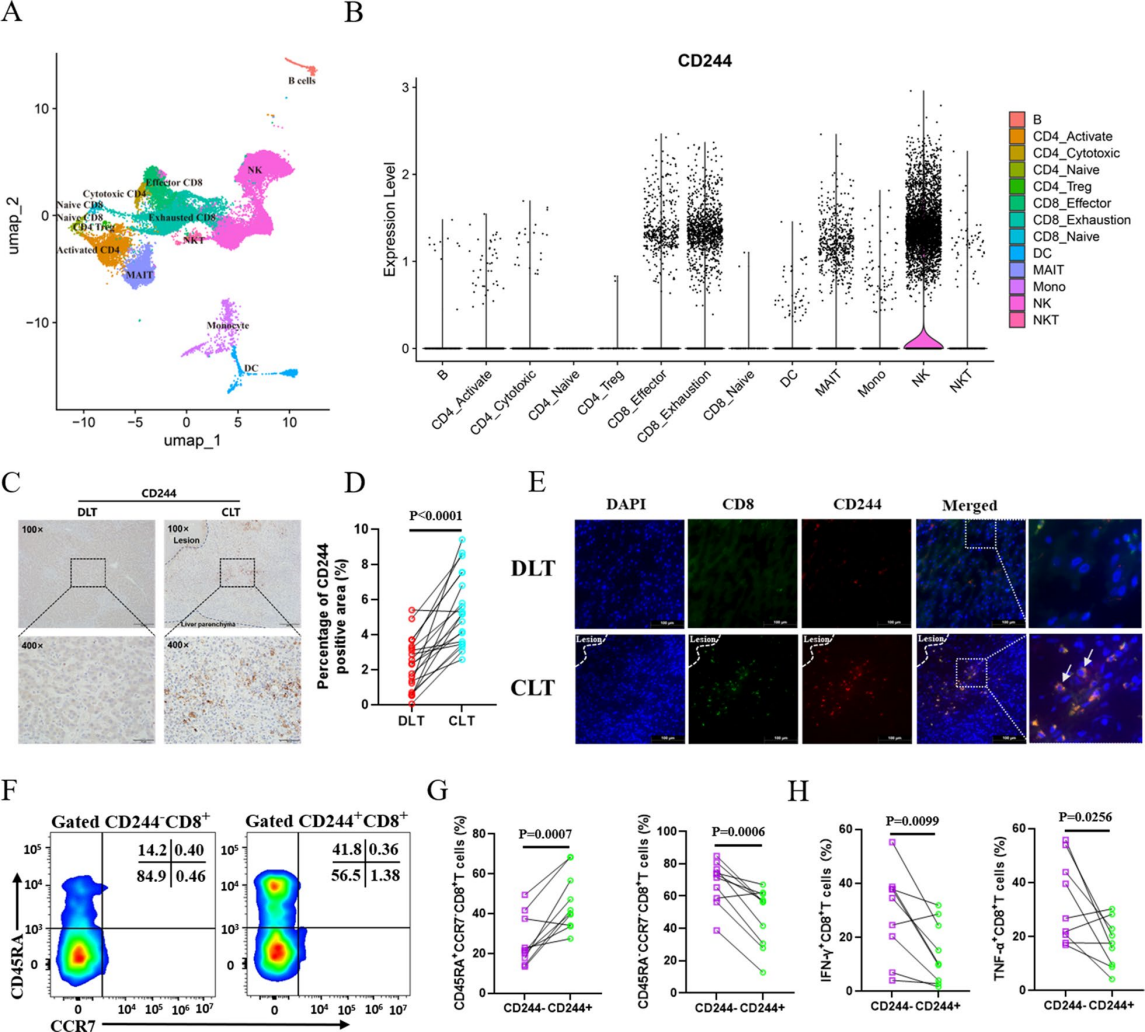
CD244 contributes to CD8^+^ T cell exhaustion in patients with hepatic AE. **A** Visualization of various immune cell subtypes using Uniform Manifold Approximation and Projection (UMAP) based on single-cell RNA sequencing data from the CLT of patients with AE (number of patients = 4, total cell count = 29,369, accession no. HRA000553). **B** The expression levels of CD244 in various immune cell types. **C** Representative immunohistochemical staining of CD244 in liver tissue sections from patients with AE is displayed (top panel 100×, bottom panel enlarged to 400×). **D** The percentage of positive staining area was calculated to evaluate the expression of CD244 (*n* = 20). **E** Representative images from immunofluorescence co-staining of DAPI (blue), CD8 (green), CD244 (red) and a merged image on the liver tissue sections from patients with AE (*n* = 6). Boxed areas show ×400 magnification of histological images. Arrows indicate CD244^+^CD8^+^ T cells. Lesions are delimited with a white dashed line. **F**, **G** FCM analysis of differentiation phenotypes of CD244^−^CD8^+^ T cells and CD244^+^CD8^+^ T cells in the CLT of patients with AE. **H** FCM analysis of the ability of CD244^−^CD8^+^ T cells and CD244^+^CD8^+^ T cells in the CLT of AE patients to secrete IFN-γ and TNF-α. CLT, “close” liver tissue; DLT, “distant” liver tissue. Data were analyzed using paired Student’s t-tests. All data are presented as mean ± SD

Single-cell sequencing analysis of CLT in hepatic AE patients showed that CD244 was predominantly expressed in clusters of NK cells and CD8^+^ T cell clusters. The proportion of exhausted CD8^+^ T cells was higher than that of effector CD8^+^ T cells (21.20% vs 7.71%) (Fig. 1A). Therefore, the expression ratio of CD244 in exhausted CD8^+^ and effector CD8^+^ T cells is roughly equal (16.80% vs. 17.87%), but there are more CD244^+^ exhausted CD8^+^ T cells (Fig. 1B).

Subsequently, immunohistochemical staining was used to assess CD244 protein expression in liver tissues from AE patients, revealing a marked increase in CD244^+^ cells in CLT compared to DLT samples (Fig. 1C, D). Furthermore, the expression level of CD244 in CLTs seemed to exhibit a weak positive correlation with the local staging of the lesions (Fig. S1D), suggesting that CD244 expression levels may predict AE progression to some extent. However, no significant correlation was found between the proportion of CD244 expression and liver enzymes (Fig. S1A–C). Additionally, through frozen sections and multiplex immunofluorescence staining, colocalization of CD244 with CD8 was observed, indicating significant recruitment of CD244^+^CD8^+^T cells in CLT of AE patients (Fig. 1E). FCM analysis revealed that, compared to CD244^−^CD8^+^ T cells, CD244^+^CD8^+^ T cells had a higher proportion of the terminally differentiated effector memory phenotype, with a marked decrease in their ability to secrete TNF-α and IFN-γ (Fig. 1F–H, Fig. S1E). Taken together, these findings suggest that upregulation of CD244 may contribute to the terminal differentiation and exhaustion of CD8^+^ T cells in hepatic AE.

### CD244^+^CD8^+^ T cells in WT mice with *E. multilocularis* infection show terminal differentiation and exhaustion phenotypes

**Fig. 2:**
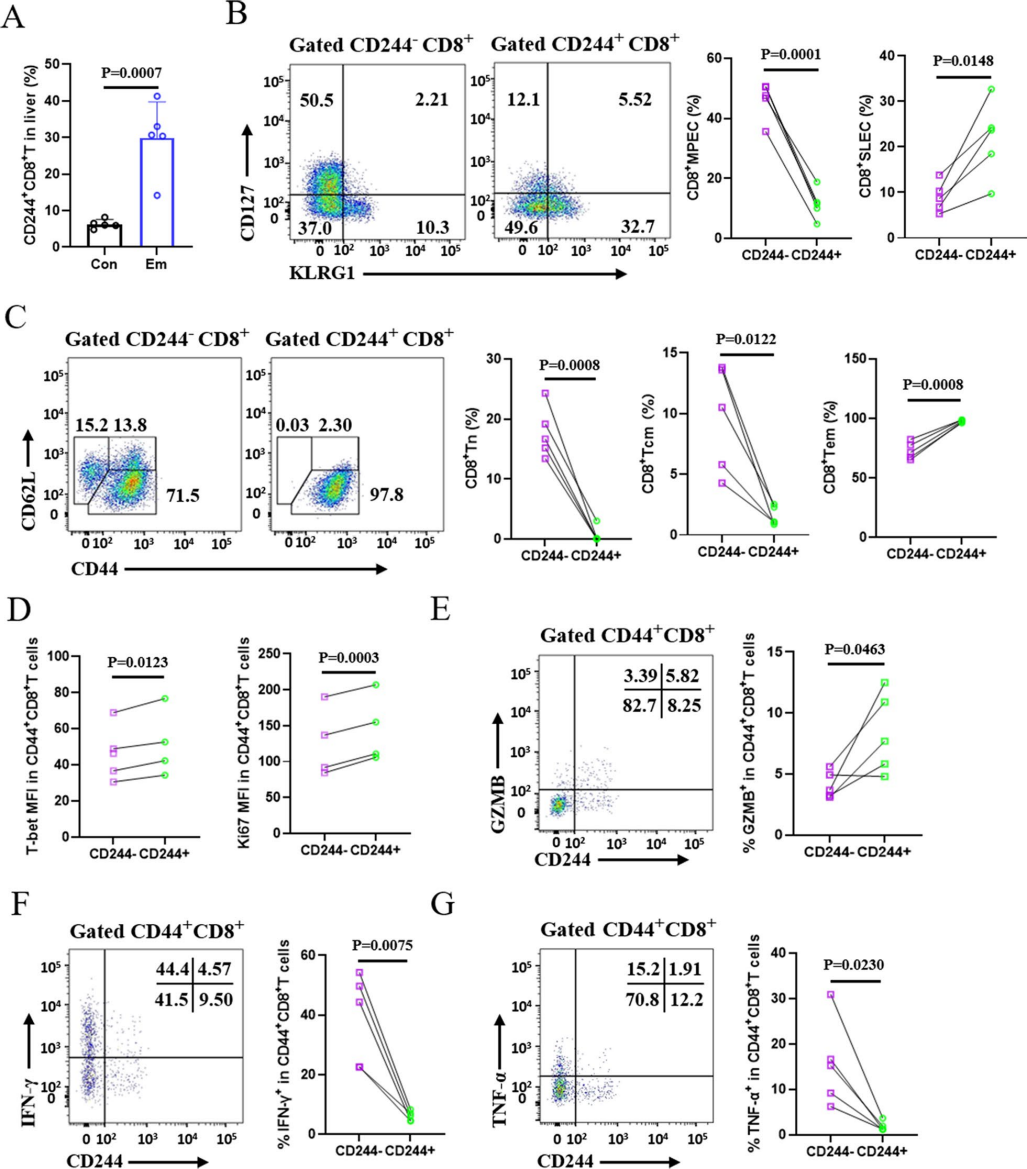
High expression of CD244 promotes the terminal differentiation effector phenotype of CD8^+^ T cells in liver of *Echinococcus multilocularis*-infected mice after 24 weeks of infection. **A** Expression changes of CD244 on the CD8^+^T cells in the liver of mice infected with *E. multilocularis*. **B** Representative flow cytometry plots (left) and the frequency (right) of SLECs and MPECs in hepatic CD244^−^ or CD244^+^CD8^+^ T cells from mice after 24 weeks of infection (5 mice per group). **C** Representative flow cytometry plots (left) and the frequency (right) of CD8^+^Tn, CD8^+^Tcm and CD8^+^Tem by CD244^−^ or CD244^+^CD8^+^ T cells in the livers from mice after 24 weeks of infection (5 mice per group). **D** Mean fluorescence intensity (MFI) of T-bet and Ki67 expression by activated CD244^−^ or CD244^+^CD8^+^ T cells in the liver from mice after 24 weeks of infection (4 mice per group). **E**–**G** Representative flow cytometry plots (left) and the frequency (right) of GZMB, IFN-γ and TNF-α production by activated CD244^−^ or CD244^+^CD8^+^ T cells in the liver from mice after 24 weeks of infection (5 mice per group). Data are one representative of two independent experiments. Con, control; Em, *E. multilocularis*; KLRG1, killer cell lectin-like receptor G1. Data were analyzed using two independent samples t-tests or paired Student’s t-tests. All data are presented as mean ± SD

We initially examined the expression of CD244 on CD8^+^ T cells in the liver tissues of WT mice infected with *E. multilocularis* for 24 weeks. The results indicated a significant increase in CD244 expression on the surface of CD8^+^ T cells in the liver of infected mice compared to uninfected controls, although this increase was not observed in the spleen (Fig. 2A, Fig. S2A). Subsequent analyses were conducted to assess the impact of CD244 on CD8^+^ T cell differentiation. First, we used CD127 and KLRG1 to define the differentiation stages of CD8^+^ T cells. As reported in the literature [24], high expression of CD127 is typically regarded as a marker of memory T cells, while high expression of KLRG1 reflects that T cells are in a terminally differentiated effector state. In our study, compared to CD244^−^CD8^+^ T cells, a higher proportion of hepatic and splenic CD244^+^CD8^+^ T cells from WT infected mice were identified as terminally differentiated short-lived effector cells (SLECs, marked as KLRG1^+^CD127^−^), while the proportion of long-lived memory precursor effector cells (MPECs, marked as KLRG1^−^CD127^+^) was reduced (Fig. 2B, Fig. S2B). Additionally, using CD44 and CD62L to define CD8^+^ T cell differentiation, we found that > 80% of hepatic and splenic CD244^+^CD8^+^ T cells presented as effector memory CD8^+^ T cells (Tem, marked as CD44^+^CD62L^−^). Conversely, the proportion of central memory CD8^+^T cells (Tcm, marked as CD44^+^CD62L^+^) among CD244^+^CD8^+^ T cells was lower, comprising < 3% in the liver and < 15% in the spleen (Fig. 2C, Fig. S2C).

We also assessed the expression of the transcription factor T-box expressed in T cell (T-bet), which is crucial for the maintenance of exhausted T cells and is highly expressed in precursor exhausted CD8^+^ T cells [25, 26]. The results showed that in the liver and spleen, the expression level of T-bet was higher in CD244^+^CD8^+^ T cells compared to CD244^−^CD8^+^ T cells, suggesting that CD244 may promote the differentiation of precursor exhausted CD8^+^ T cells (Fig. 2D, Fig. S2D). Interestingly, when we assessed the proliferation marker Ki67, we found that in the liver and spleen, the proportion of Ki67-expressing CD8^+^ T cells was higher in the CD244-positive group compared to the CD244-negative group (Fig. 2D, Fig. S2D). This may be due to the fact that half of the cells in the CD244-positive group are effector CD8^+^ T cells, while the other half belong to exhausted CD8^+^ T cells. Both the effector CD8^+^ T cells and the precursor within the exhausted CD8^+^ T cell population exhibit a strong proliferative capacity [27].

Furthermore, we assessed the effector function of CD8^+^ T cells and found that, compared to CD244^−^ cells, CD244^+^CD8^+^ T cells from both the liver and spleen exhibited a noticeable reduction in the production of IFN-γ and TNF-α. However, hepatic CD244^+^CD8^+^ T cells showed a significant increase in Granzyme B (GZMB) production (Fig. 2E–G, Fig. S2E–G). Collectively, these findings demonstrate that CD244^+^CD8^+^ T cells display terminal differentiation and exhaustion phenotypes in mice with advanced *E. multilocularis* infection.

### CD244 deficiency enhances the cytotoxic function of mouse hepatic CD8^+^ T cells in vitro

**Fig. 3:**
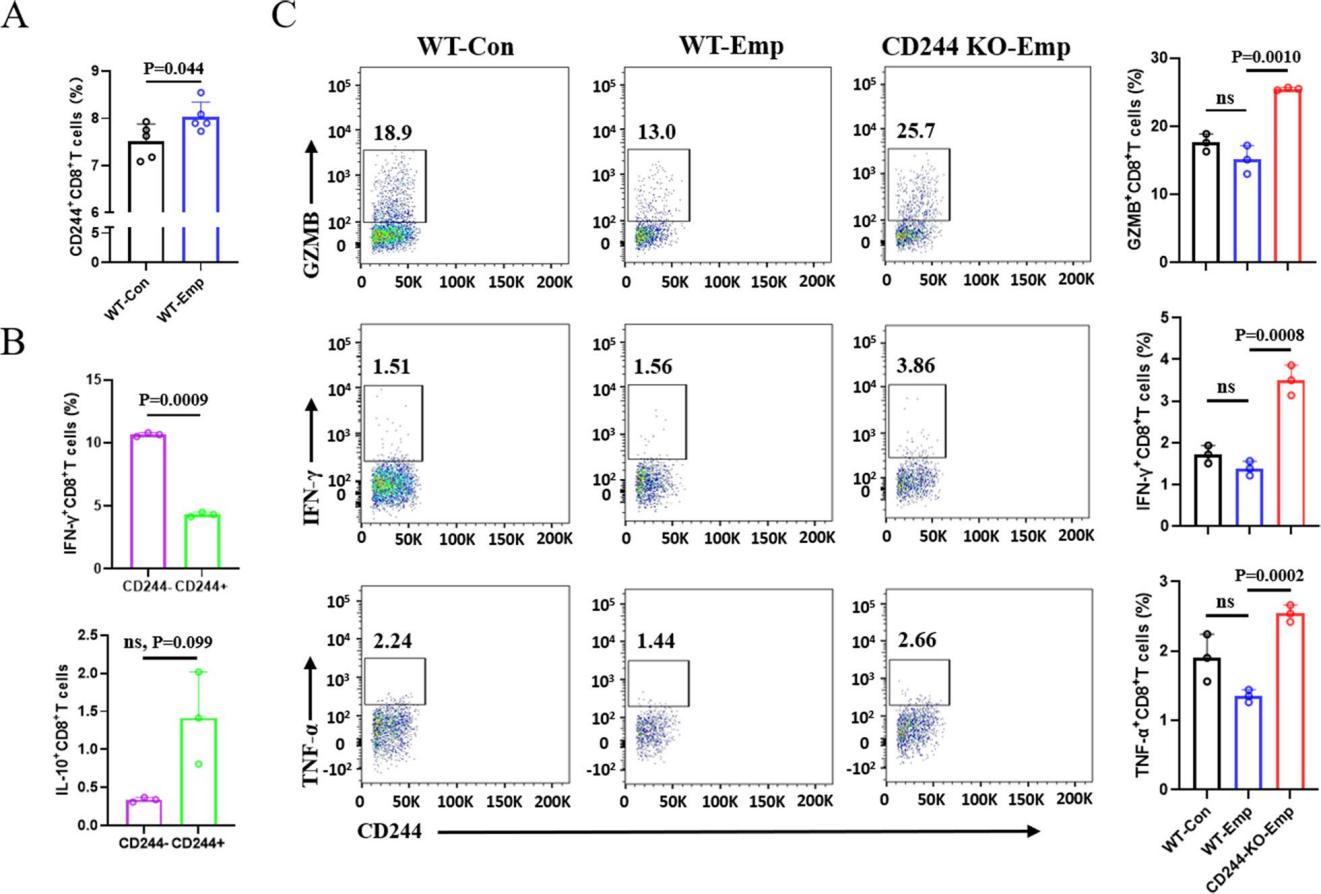
CD244 induces functional exhaustion in hepatic CD8^+^ T cells from mice exposed to *Echinococcus multilocularis* protoscoleces antigens in vitro. **A** FCM detection of CD244 expression on hepatic CD8^+^T cells from WT mice after in vitro stimulation with *E. multilocularis* protoscoleces antigen (Emp) for 48 h. **B** FCM analysis of changes in the secretion capacity of the pro-inflammatory cytokine IFN-γ and the inhibitory cytokine IL-10 by CD244^−^CD8^+^ T cells and CD244^+^CD8^+^ T cells in mouse liver following Emp stimulation for 48 h. **C** Representative flow cytometry plots (left) and the frequencies (right) of Granzyme-B, IFN-γ and TNF-α production by hepatic CD8^+^ T cells from control, WT and CD244-KO mice with Emp stimulation for 48 h in vitro. Data are one representative of two independent experiments. Con, control; Em, *E. multilocularis*; KO, knockout; WT, wild type; Emp, *E. multilocularis* protoscoleces. Data were analyzed using paired Student’s t-tests or two independent samples t-tests. All data are presented as mean ± SD. ns, *P* > 0.05

To investigate whether CD244 affects the cytotoxic function of CD8^+^ T cells, we initially stimulated liver lymphocytes from WT mice with *E. multilocularis* protoscoleces (Emp) antigen for 48 h in vitro. The results showed significant upregulation of CD244 expression on the surface of CD8^+^ T cells in WT mice compared to the control group (Fig. 3A). Additionally, there was a notable decrease in IFN-γ secretion ability and an increase in IL-10 secretion by CD244^+^CD8^+^ T cells, suggesting that elevated CD244 expression mediated immune tolerance in CD8^+^ T cells (Fig. 3B). We then stimulated liver lymphocytes from both WT and CD244-KO mice with Emp in vitro. The results demonstrated a significant enhancement in the ability of CD244-deficient CD8^+^ T cells to secrete GZMB, INF-γ and TNF-α (Fig. 3C). However, no significant changes were observed in the cytokine secretion capacity of NK cells and CD4^+^ T cells (Fig. S3). Interestingly, hepatic NK cells from CD244-KO mice exhibited remarkably enhanced ability to secrete GZMB.

### Reversal of CD8^+^ T cell exhaustion by CD244 deficiency decelerates the progression of *E. multilocularis*

**Fig. 4:**
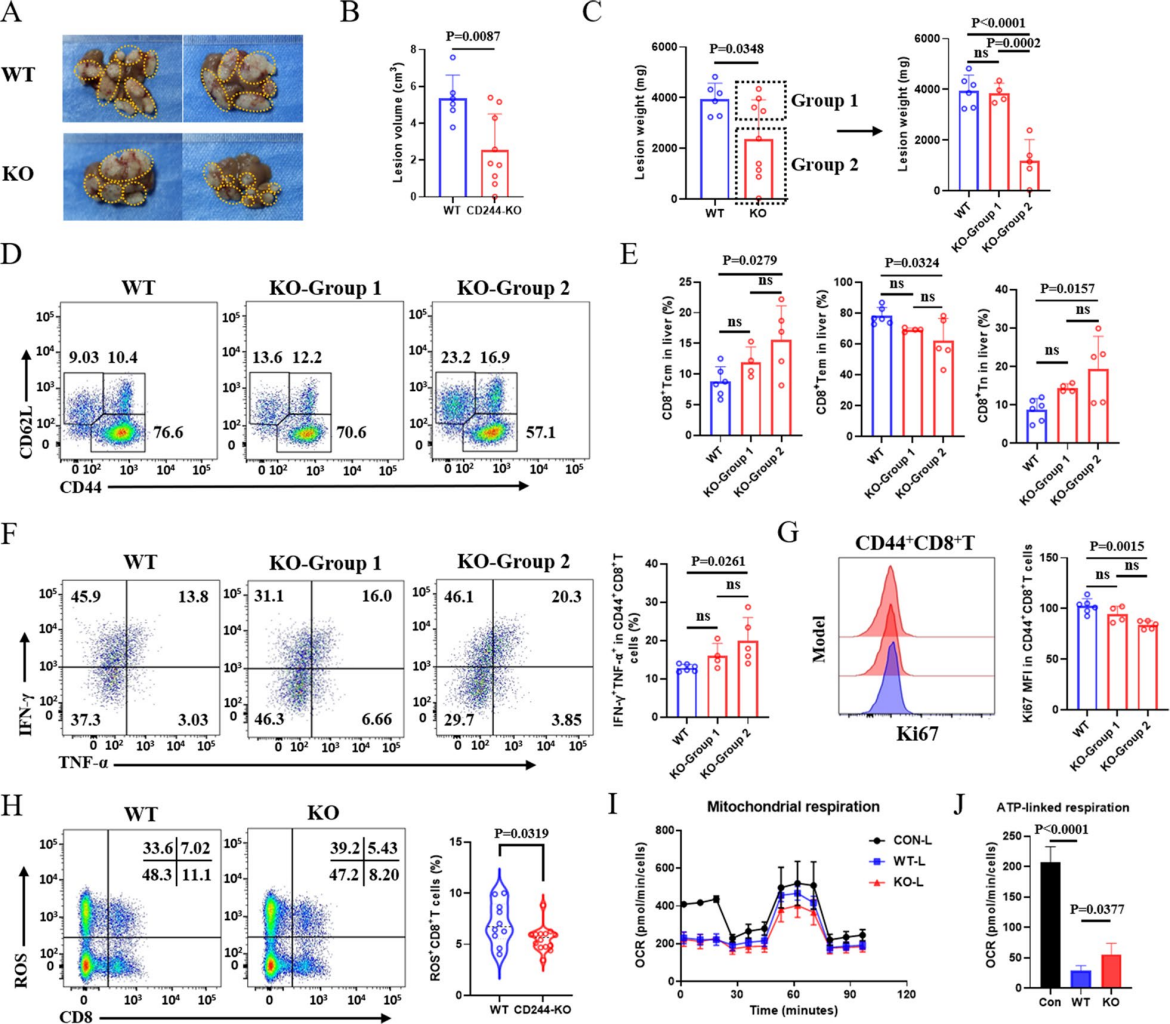
CD244 deficiency delays disease progression by inhibiting terminal differentiation and functional exhaustion of CD8^+^ T cells in the liver of *Echinococcus multilocularis*-infected mice after 19 weeks of infection. **A** Representative images of metacestode tissue in the liver from WT and CD244-KO mice after 19 weeks of infection. The metacestode tissues are delineated with a yellow line. Owing to the heterogeneity of lesion weight, KO group mice were divided into two subgroups, named Group 1 and Group 2. **B**, **C** Lesion volume and weight in the liver of WT and CD244-KO mice after 19 weeks of infection (4–6 mice per group). **D**, **E** Representative flow cytometry plots (left) and frequency (right) of differentiation phenotypes of CD8^+^ T cells in the liver of mice at 19 weeks post-infection with *E. multilocularis* (4–6 mice per group). **F** Representative flow cytometry plot and percentage of IFN-γ and TNF-α production by activated CD8^+^ T cells in the liver from mice after 19 weeks of infection (4–6 mice per group). **G** MFI of Ki67 expression by activated CD8^+^ T cells in the liver from mice after 19 weeks of infection (4–6 mice per group). **H** Flow cytometry assessment of hepatic ROS^+^CD8^+^ T cells from CD244-KO and WT mice after 19 weeks of *E. multilocularis* infection (10–14 mice per group). **I** Oxygen consumption rate (OCR) of hepatic CD8^+^ T cells from *E. multilocularis*-infected mice was measured following treatment with oligomycin, FCCP and rotenone plus antimycin A (Rot/AA). **J** Statistical analysis of mitochondrial ATP-linked respiration in hepatic CD8^+^ T cells from mice infected with *E. multilocularis*. Data are one representative of two independent experiments. KO, knockout; WT, wild type. Data were analyzed using two independent samples t-tests, Kruskal-Wallis test or one-way ANOVA test. ns, *P* > 0.05

To further substantiate the involvement of CD244 in the exhaustion of CD8^+^ T cells during *E. multilocularis* infection, we investigated WT and CD244-KO mice infected for 19 weeks. Interestingly, while the overall lesion load in CD244-deficient mice was significantly reduced, only half of the CD244-KO mice (Group 2) exhibited a noticeable decrease in lesion weight, whereas the lesions in the other half (Group 1) were not significantly different from those in the WT group (Fig. 4A–C). We also examined the activation phenotype of CD8^+^ T cells. Results showed that, compared to WT mice, CD244-KO mice in Group 2 had a significant increase in the proportion of central memory CD8^+^ T cells and a significant decrease in the proportion of effector memory CD8^+^ T cells within both liver and spleen CD8^+^ T cell populations; however, CD244-KO mice in Group 1 only exhibited a trend towards these changes (Fig. 4D, E; Fig. S4A, B).

Interestingly, compared to WT mice, the proportion of CD8^+^ T cells capable of secreting multifunctional cytokines (IFN-γ^+^TNF-α^+^) in the liver and spleen of CD244-KO mouse groups was significantly increased (Fig. 4F, Fig. S4C). However, the expression level of Ki67 was significantly reduced in the hepatic CD8^+^ T cells of CD244-KO mice, but there was no significant change in the spleen (Fig. 4G, Fig. S4D). In summary, these findings suggest that CD244 is involved in mediating CD8^+^ T cell exhaustion. The deficiency of CD244 inhibits the terminal effector differentiation of CD8^+^ T cells while promoting the formation of central memory CD8^+^ T cells and concurrently restores the overall cytotoxic effector function of CD8^+^ T cells.

To explore the mechanism of CD244-mediated CD8^+^ T cell exhaustion, we assessed intracellular reactive oxygen species (ROS) levels in hepatic and splenic CD8^+^ T cells from mice with *E. multilocularis* infection. It is well known that excessive ROS is an important inducer of T cell exhaustion [28]. The findings show that compared to wild-type (WT) mice, the levels of ROS in CD8^+^ T cells in the liver and spleen of CD244-KO mice were significantly reduced (Fig. 4H, Fig. S4E). Furthermore, we used a Seahorse extracellular flux analyzer to measure the OCR of CD8^+^ T cells derived from the liver and spleen. The results indicated that, compared to the control group mice, mitochondrial respiration associated with ATP synthesis (ATP-linked respiration) in CD8^+^ T cells from the liver and spleen of WT-infected mice was significantly reduced. In contrast, CD244 deficiency specifically enhanced mitochondrial ATP-linked respiration in hepatic CD8^+^ T cells from mice infected with *E. multilocularis* (Fig. 4I, J, Fig. S4F, G). These results suggest a potential role for targeting CD244 to improve mitochondrial function in hepatic CD8^+^ T cells during *E. multilocularis* infection, thereby reversing the exhaustion state of CD8^+^ T cells.

### Adoptively transferred CD244^−/−^CD8^+^ T cells exhibit enhanced effector function in *E. multilocularis*-infected CD8-KO mice

**Fig. 5:**
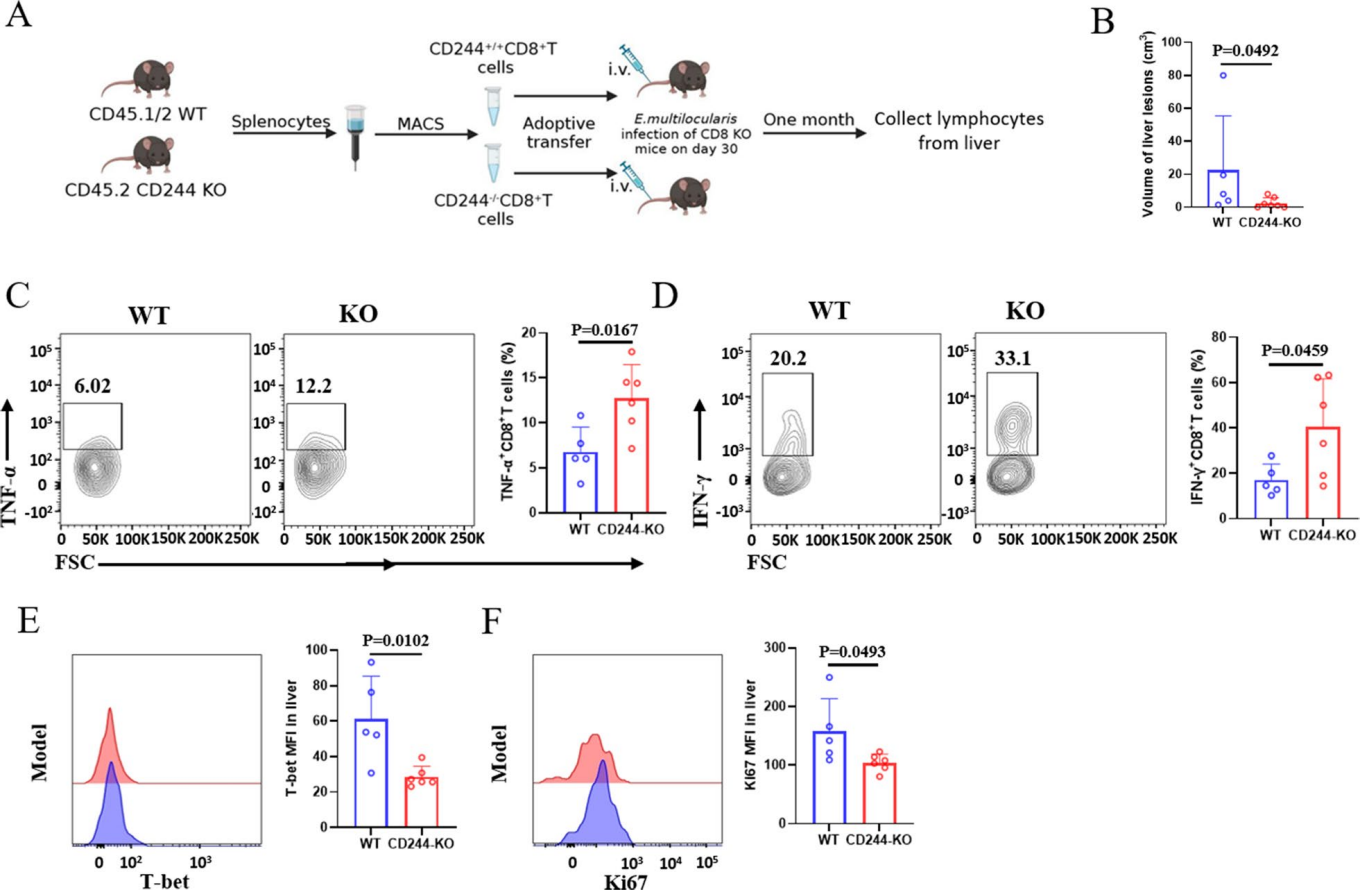
CD244-deficient CD8^+^ T cells demonstrate enhanced effector function following adoptive transfer into *Echinococcus multilocularis*-infected CD8-KO mice. **A** Establishment of the adoptive transfer mouse model: CD8^+^ T cells were isolated and purified from the spleens of CD244-KO (CD45.2) and WT mice (CD45.1/2) and transferred into CD8-KO recipient mice 30 days post-*E. multilocularis* infection (5–7 mice per group). **B** Lesion volume in the livers of two groups of CD8-KO mice 30 days after adoptive transfer. **C**, **D** Representative flow cytometry plots and percentages of TNF-α and IFN-γ production by WT and CD244-KO donor CD8^+^ T cells in the livers of *E. multilocularis*-infected CD8-KO recipient mice. **E**, **F** Representative flow cytometry plots and mean fluorescence intensity (MFI) of T-bet and Ki67 expression by transferred CD8^+^ T cells in the livers of *E. multilocularis*-infected CD8-KO mice. KO, knockout; WT, wild type. Data were analyzed using two independent samples t-test or Mann-Whitney test

To further investigate whether CD244 is necessary for mediating and maintaining CD8^+^ T cell exhaustion induced by *E. multilocularis* infection, we isolated and purified CD8^+^ T cells from the spleens of both WT and CD244-deficient mice. These cells were then transferred via tail vein injection to CD8-KO mice infected with *E. multilocularis* for 30 days. After 30 days post-transfer, we isolated liver lymphocytes from the CD8-KO recipient mice and performed flow cytometry analysis to assess the functionality of CD8^+^ T cells from WT and CD244-KO donors (Fig. 5A). Although no significant differences were observed in the proportions and absolute numbers of total CD8^+^ T cells, CD8^+^ Tcm, CD8^+^ Tem and CD8^+^ Tn cells between the liver of the donor mice (Fig. S5), the ability of CD244-KO donor CD8^+^ T cells to secrete IFN-γ and TNF-α was significantly enhanced in the liver (Fig. 5C, D). Correspondingly, the transfer of CD244-KO donor CD8^+^ T cells resulted in a significant reduction in the volume of liver lesions in CD8-KO recipient mice (Fig. 5B). Interestingly, compared to WT donor CD8^+^ T cells, transferred CD244-deficient CD8^+^ T cells in liver exhibited decreased expression of T-bet and Ki67 (Fig. 5E, F). These results highlighted that CD244 deficiency in CD8^+^ T cells inhibited their terminal exhaustion differentiation and enhanced their effector function during *E. multilocularis* infection.

## Discussion

The immune tolerance characteristics of the liver make it a primary target organ for invasion and infection by various parasites [29]. Previous research has demonstrated that dysfunction or deficiency of CD8^+^ T cells exacerbates liver lesions in mice infected with *E. multilocularis*, underscoring the vital role of CD8^+^ T cells in combating parasitic infections. In the CLT of AE patients, although CD8^+^ T cells were significantly recruited, their cytokine secretion capacity was markedly diminished compared to that in the DLT, exhibiting an exhausted phenotype [12]. These findings suggest that effectively restoring the functionality of CD8^+^ T cells may represent a novel immunotherapeutic strategy for AE.

Persistent parasitic infection can induce T-cell exhaustion through the modulation of co-inhibitory receptor expression [30]. In a previous study, we observed that CD8^+^ T cells with high expression of the inhibitory receptor CD244 in the livers of *E. multilocularis*-infected mice exhibited an exhausted phenotype [22]. However, the specific details on how CD244 mediates CD8^+^ T cell exhaustion remains unclear. Here, we have observed that CD244 facilitates the induction of terminal effector differentiation programs of CD8^+^ T cells, ultimately leading to their exhaustion. This suggests that targeting the immune checkpoint CD244 could potentially serve as a novel immunotherapeutic strategy for AE.

In fact, similar to acute infections, in the early stages of *E. multilocularis* infection, CD8^+^ T cells differentiate into SLEC, which are capable of effectively clearing the pathogens [31]. However, under persistent antigen presence, these effector T cells ultimately transition into a state of terminal exhaustion. Early studies have indicated that CD244 is involved in the differentiation and development of T cells during chronic infections [32], where it is closely associated with the exhaustion of CD8^+^ T cells [33, 34]. For instance, in studies of chronic HBV infection, it has been confirmed that the high expression of CD244 on the surface of terminally differentiated effector CD8^+^ T cells exerts an inhibitory function. Blocking CD244 can enhance the ability of CD8^+^ T cells to secrete cytokines [35]. In research on tumor diseases, it has also been found that CD244 is highly expressed on the surface of T cells in a mouse model of lung cancer, inhibiting the cytokine secretion capacity of T cells while promoting T cell apoptosis, leading to T cell exhaustion [36]. Furthermore, researchers have observed an increased proportion of terminally differentiated effector memory CD8^+^ T cells in patients with active leishmaniasis, which also exhibit high expression of the inhibitory receptors CD160 and CD244 on their surface, indicating a state of functional exhaustion [37].

In accordance with the aforementioned findings, our study found that CD244 mediated the terminal effector differentiation of CD8^+^ T cells, thereby accelerating their functional exhaustion. Knocking out CD244 not only inhibited the generation of CD8^+^ Tem cells in the liver and spleen of mice infected with *E. multilocularis* but also promoted the development of CD8^+^ Tcm cells and enhanced the ability to secrete multifunctional cytokines (IFN-γ^+^TNF-α^+^) of CD8^+^ T cells in the liver and spleen of mice with *E.* *multilocularis*, leading to a substantial reduction in parasite load. These findings suggest that targeting CD244 in CD8^+^ T cells could potentially offer a new opportunity for immunotherapy in AE. However, surprisingly, although the knockout of CD244 improved the functional exhaustion of hepatic CD8^+^ T cells, the expression level of the proliferation marker Ki67 significantly decreased. This result suggests that CD244 may have different regulatory mechanisms for the effector function and proliferative capacity of CD8^+^ T cells.

Moreover, we observed that changes in transcriptional regulation do not appear to align with the functional alterations in CD8^+^ T cells. It is commonly understood that T-bet regulates the cytotoxicity of CD8^+^ T cells and the production of IFN-γ [38]. In fact, CD8^+^ T cells can secrete IFN-γ through both T-bet-dependent and T-bet-independent pathways [39, 40]. In this study, we found that in the livers of mice infected with *E. multilocularis*, the expression of T-bet in exhausted CD244-positive CD8^+^ T cells was higher than that in the CD244-negative group. However, when CD244-deficient splenic CD8^+^ T cells were transferred into CD8-KO mice infected with *E. multilocularis*, the functionality of the infiltrating CD244^−/−^ CD8^+^ T cells in the liver was significantly enhanced, while the expression of T-bet was markedly reduced. This suggests that the enhancement of CD8^+^ T cell functionality in the livers of infected mice through the knockout of CD244 may not depend on the expression of T-bet. Furthermore, the transcription factors T-bet play crucial roles in orchestrating the terminal differentiation of CD8^+^ T cells [25, 41]. Our data confirm that, compared to the CD244-negative group, the expression of T-bet is elevated in the CD244-positive group, and a greater proportion of CD244-positive CD8^+^ T cells differentiates into terminally differentiated SLECs. However, in the adoptive transfer experiments, there were no significant differences in the proportion and number of CD8^+^ Tem cells, which may be attributed to the limited number of transferred cells and the small sample size, warranting further investigation.

In addition, it is worth noting that after persistent antigen stimulation, T cell mitochondria produce excess ROS during glucose metabolism [42]. Studies have shown that excessive mitochondrial ROS can sustain activation of the Nuclear Factor of Activated T cells (NFAT) signaling in T cells, thereby driving terminal exhaustion [43]. Our data indicated that though knocking out CD244 reduced the ROS levels in CD8^+^ T cells in the liver of *E. multilocularis*-infected mice. Additionally, CD244 deficiency improved mitochondrial ATP-linked respiration specifically in hepatic CD8^+^ T cells from these mice, suggesting an improvement in mitochondrial metabolic status. These results suggest that targeting CD244 may facilitate metabolic reprogramming of CD8^+^ T cells, which is significant for the development of new therapeutic strategies.

## Conclusions

In summary, our study confirms that the upregulation of CD244 is involved in mediating immune exhaustion of CD8^+^ T cells in patients with AE and in late-stage WT mice infected with *E. multilocularis*. Our research results provide a theoretical basis for further studies on targeting immune checkpoints to reverse states of T cell exhaustion.

## Supplementary Information

**Additional file 1: Table S1.** Baseline clinical characteristics of AE patients studied.

**Additional file 2: Table S2. **Liver samples from AE patients used for immunological studies.

**Additional file 3: Table S3.** Antibodies for flow cytometry.

**Additional file 4: Fig. S1.** Correlation analysis between the proportion of CD244 positivity and the levels of AST, ALT, ALP and local lesion staging.

**Additional file 5: Fig. S2.** CD244 enhances the terminal differentiation and effector phenotype of CD8^+^ T cells in the spleens of *Echinococcus multilocularis*-infected mice after 24 weeks of infection.

**Additional file 6: Fig. S3.** FCM assessed changes in the secretion capacities of GZMB, IFN-γ, TNF-α and IL-10 by liver NK cells and CD4^+^ T cells from control, WT and CD244-KO mice following 48 h of in vitro Emp stimulation.

**Additional file 7: Fig. S4.** CD244 deficiency prevents terminal differentiation and functional exhaustion of CD8^+^ T cells in the spleens of *Echinococcus multilocularis*-infected mice at 19 weeks post-infection.

**Additional file 8: Fig. S5.** CD244 deficiency does not significantly affect the differentiation of donor CD8^+^ T cells following adoptive transfer into *Echinococcus multilocularis*-infected CD8-KO mice.

**Additional file 9:** **Fig. S6.** Flow cytometric gating strategies.

## Data Availability

No datasets were generated or analysed during the current study.

## Abbreviations

AE: Alveolar echinococcosis
CLT: Close liver tissue
DLT: Distant liver tissue
ROS: Reactive oxygen species
OCR: Oxygen consumption rate
ATP: Adenosine triphosphate
*E. multilocularis*: *Echinococcus multilocularis*
WHO: World Health Organization
IRs: Inhibitory receptors
IL-10: Interleukin-10
TGF-β: Transforming growth factor beta
PD-1: Programmed cell death 1
TIGIT: T cell immunoreceptor with immunoglobulin and immunoreceptor tyrosine-based inhibitory motif domain
SLAM: Signaling lymphocyte activation molecule
MDSCs: Myeloid-derived suppressor cells
SAP: SLAM associated protein
EAT2: Ewing sarcoma-activated transcript 2
LCMV: Lymphocytic choriomeningitis virus
IFN-γ: Interferon-gamma
TNF-α: Tumor necrosis factor-alpha
PSCs: *E. multilocularis* Protoscoleces
PBS: Phosphate buffer saline
BSA: Bovine serum albumin
WT: Wild type
KO: Knockout
FCM: Flow cytometry
SLECs: Short-lived effector cells
MPECs: Memory precursor effector cells
Tem: Effector memory T cells
Tcm: Central memory T cells
T-bet: T-box expressed in T cell
GZMB: Granzyme B
NFAT: Nuclear factor of activated T cells
