# Supplementary material for: Upregulation of CD244 promotes CD8+ T cell exhaustion in patients with alveolar echinococcosis and a murine model
Source: Parasit Vectors. 2024 Nov 23;17:483. doi: 10.1186/s13071-024-06573-2 (PMC11585139; doi:10.1186/s13071-024-06573-2)
Supplement: Supplementary file 1 — Additional file 1: Table S1. Baseline clinical characteristics of AE patients studied. [file 13071_2024_6573_MOESM1_ESM.docx]

| Paitent | Gender | Age | PNM stage | Lesion size(cm) | AST(U/L) | ALT(U/L) | Surgical procedure |
| --- | --- | --- | --- | --- | --- | --- | --- |
| 1 | F | 15 | P1N0M0 | 9.48*6.99 | 22.59 | 15.85 | RHR |
| 2 | F | 25 | P2N1M0 | 8.96*7.09 | 34.04 | 28.72 | RHR |
| 3 | F | 36 | P4N0M1 | 26.8*14.6 | 71.2 | 46.62 | RHR |
| 4 | F | 36 | P1N0M0 | 5.73*7.71 | 28.95 | 32.8 | RHR |
| 5 | F | 36 | P4N0M0 | 10.09*9.96 | 53.05 | 56.86 | ELRA |
| 6 | F | 25 | P4N0M1 | 14.60*10.90 | 52.36 | 66.73 | ELRA |
| 7 | F | 31 | P3N0M0 | 5.96*5.16 | 34.72 | 31.57 | ELRA |
| 8 | F | 54 | P3N1M0 | 15.60*10.05 | 36.3 | 33.75 | RHR |
| 9 | M | 33 | P4N1M1 | 14.05*12.68 | 33.1 | 32.9 | ELRA |
| 10 | F | 47 | P4N1M0 | 11.4*8.19 | 56.67 | 55.78 | ELRA |
| 11 | F | 28 | P3N1M0 | 9.16*7.64 | 22.82 | 24.27 | RHR |
| 12 | F | 26 | P4N0M0 | 10.17*8.62 | 64.74 | 37.85 | ELRA |
| 13 | F | 44 | P2N0M0 | 11.52*6.82 | 34.94 | 30.11 | RHR |
| 14 | M | 61 | P2N1M0 | 13.86*10.89 | 25.06 | 15 | RHR |
| 15 | M | 34 | P2N0M0 | 11.55*9.55 | 25.36 | 15 | RHR |
| 16 | M | 25 | P4N0M0 | 9.99*8.99 | 21.36 | 15 | RHR |
| 17 | M | 36 | P4N0M0 | 4.7*3.1 | 85.87 | 169 | ELRA |
| 18 | M | 19 | P4N0M0 | 10.53*6.47 | 32.05 | 36 | RHR |
| 19 | F | 34 | P3N0M1 | 8.80*5.82 | 31.15 | 27 | RHR |
| 20 | F | 52 | P4N0M1 | 13.37*13.06 | 32.84 | 18 | ELRA |

**Table S1 Baseline clinical characteristics of AE patients studied**

**Abbreviations:** F, female; M, male; P: parasitic mass in the liver; N: involvement of neighbouring organs; M: metastasis; AST: Aspartate Aminotransferase; ALT: Alanine Aminotransferase; RHR, Radical hepatic resection; ELRA: *Ex vivo* liver resection and autotransplantation.
