# Supplementary material for: Upregulation of CD244 promotes CD8+ T cell exhaustion in patients with alveolar echinococcosis and a murine model
Source: Parasit Vectors. 2024 Nov 23;17:483. doi: 10.1186/s13071-024-06573-2 (PMC11585139; doi:10.1186/s13071-024-06573-2)
Supplement: Supplementary file 2 — Additional file 2: Table S2. Liver samples from AE patients used for immunological studies. [file 13071_2024_6573_MOESM2_ESM.docx]

|  |  |  | Patients with AE | | | |
| --- | --- | --- | --- | --- | --- | --- |
| Experiment |  |  | CLT, # of Samples | DLT, # of Samples | Paired CLT and DLT,  # of Sample Pairs | Comparison Between Groups |
| Liver | IHC | CD244 | 20 | 20 | 20 | CLT vs. DLT |
|  | IF | CD8 | 6 | 6 | 6 | CLT vs. DLT |
|  |  | CD244 | 6 | 6 | 6 | CLT vs. DLT |
|  | FC | CD8 | 9 | 9 | 9 | CD244^+^ vs CD244^-^ in CLT |

**Table S2. Liver and blood samples from AE patients used for immunological studies.**
