# Supplementary material for: Upregulation of CD244 promotes CD8+ T cell exhaustion in patients with alveolar echinococcosis and a murine model
Source: Parasit Vectors. 2024 Nov 23;17:483. doi: 10.1186/s13071-024-06573-2 (PMC11585139; doi:10.1186/s13071-024-06573-2)
Supplement: Supplementary file 3 — Additional file 3: Table S3. Antibodies for flow cytometry. [file 13071_2024_6573_MOESM3_ESM.doc]

**Table S3. Antibodies for flow cytometry**

| **Reagent or Resource** | **Clone** | **Source** | **Catalog Number** |
| --- | --- | --- | --- |
| Anti-human CD56-Alexa Fluor® 700 | 5.1H11 | BioLegend | Cat# 362521 |
| Anti-human CD3-PE/Cyanine7 | UCHT1 | BioLegend | Cat# 300419 |
| Anti-human CD3-PerCP/Cyanine5.5 | UCHT1 | BioLegend | Cat# 300429 |
| Anti-human CD3-Alexa Fluor® 700 | UCHT1 | BioLegend | Cat# 300423 |
| Anti-human CD8-PE/DazzleTM 594 | SK1 | BioLegend | Cat# 344743 |
| Anti-human CD8-Brilliant Violet 510^TM^ | SK1 | BioLegend | Cat# 344731 |
| Anti-human CD8-PE/Cyanine7 | SK1 | BioLegend | Cat# 344711 |
| Anti-human CD4-APC/Cyanine7 | RPA-T4 | BioLegend | Cat# 300517 |
| Anti-human CD45RA-Brilliant Violet 650^TM^ | HI100 | BioLegend | Cat# 304135 |
| Anti-human CCR7-Alexa Fluor® 700 | G043H7 | BioLegend | Cat# 353243 |
| Anti-human CCR7- PE/Cyanine7 | G043H7 | BioLegend | Cat# 353225 |
| Anti-human CD244-PE/Dazzle^TM^ 594 | C1.7 | BioLegend | Cat# 329521 |
| Anti-human CD244- PerCP/Cyanine5.5 | C1.7 | BioLegend | Cat# 329515 |
| Anti-human IFN-γ-APC | 4S.B3 | BioLegend | Cat# 502512 |
| Anti-human TNF-α-PerCP/Cyanine5.5 | Mab11 | BioLegend | Cat# 502926 |
| Anti-human GZMB-FITC | GB11 | BD | Cat# 560211 |
| Purified anti-mouse CD16/32 |  | BioLegend | Cat# 101302 |
| Anti-mouse NK1.1- PE/Cyanine7 | PK136 | BioLegend | Cat# 108714 |
| Anti-mouse CD3-FITC | 17A2 | BioLegend | Cat# 100204 |
| Anti-mouse CD3-Brilliant Violet 510^TM^ | 17A2 | BioLegend | Cat# 100233 |
| Anti-mouse CD8-APC/Cyanine7 | 53-6.7 | BioLegend | Cat# 100714 |
| Anti-mouse CD4- Brilliant Violet 605™ | GK1.5 | BioLegend | Cat# 100451 |
| Anti-mouse CD44-Alexa Fluor® 700 | IM7 | BioLegend | Cat# 103026 |
| Anti-mouse CD44-APC | IM7 | BioLegend | Cat# 103012 |
| Anti-mouse CD62L-Brilliant Violet 510^TM^ | MEL-14 | BioLegend | Cat# 104441 |
| Anti-mouse CD244-PE | M2B4(B6)458.1 | BioLegend | Cat# 133507 |
| Anti-mouse KLRG1- PerCP/Cyanine5.5 | 2F1/KLRG1 | BioLegend | Cat# 138417 |
| Anti-mouse CD127-APC | AFR34 | BioLegend | Cat# 135012 |
| Anti-mouse IFN-γ-APC | XMG12 | BioLegend | Cat# 505810 |
| Anti-mouse TNF-α- PerCP/Cyanine5.5 | MP6-XT22 | BioLegend | Cat# 506322 |
| Anti-mouse GZMB- Pacific Blue™ | GB11 | BioLegend | Cat# 515408 |
| Anti-mouse T-bet- PE/Dazzle^TM^ 594 | 4B10 | BioLegend | Cat# 644828 |
| Anti-mouse Ki67- PerCP/Cyanine5.5 | 16A8 | BioLegend | Cat# 652424 |
