# Supplementary material for: Upregulation of CD244 promotes CD8+ T cell exhaustion in patients with alveolar echinococcosis and a murine model
Source: Parasit Vectors. 2024 Nov 23;17:483. doi: 10.1186/s13071-024-06573-2 (PMC11585139; doi:10.1186/s13071-024-06573-2)
Supplement: Supplementary file 4 — Additional file 4: Fig. S1. Correlation analysis between the proportion of CD244 positivity and the levels of AST, ALT, ALP and local lesion staging. [file 13071_2024_6573_MOESM4_ESM.docx]

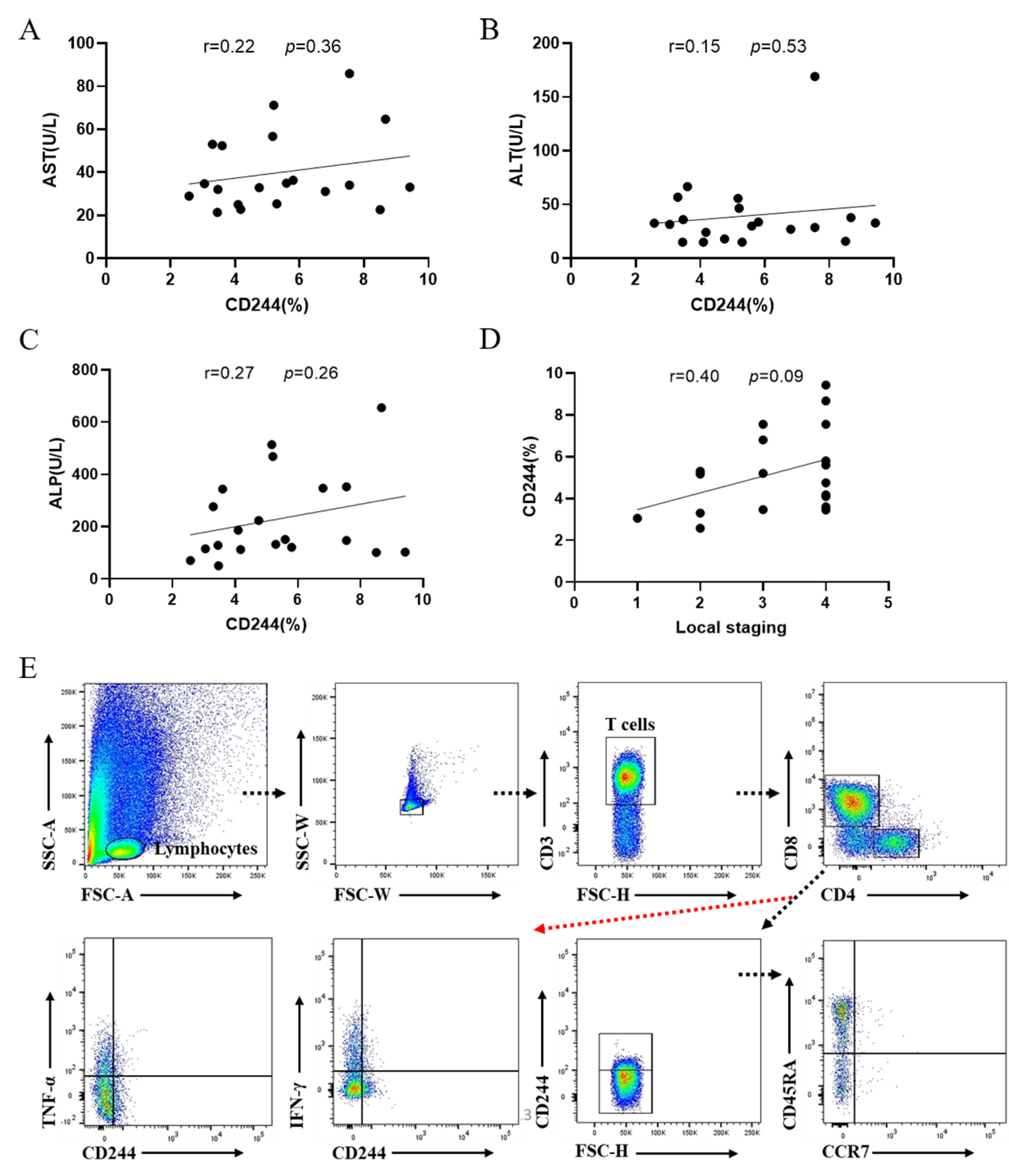


**Fig. S1. Clinical relevance analysis results and flow cytometric gating strategy.** (A-D) CD244 immunohistochemical staining was performed on the CLT of AE patients, Olympus software was used to measure the proportion of positive staining area, correlation analysis was conducted between the measurement of CD244 and the levels of AST, ALT, ALP, and local lesion staging. (E). Flow cytometric gating strategy used to identify the differentiation phenotype and functional changes of liver-infiltrating CD8^+^ T cells from AE patients. CLT, close liver tissue; AE: alveolar echinococcosis; AST, Aspartate aminotransferase; ALT, Alanine aminotransferase; ALP, Alkaline phosphatase. Data were analyzed using Pearson correlation test.
