# Supplementary material for: Upregulation of CD244 promotes CD8+ T cell exhaustion in patients with alveolar echinococcosis and a murine model
Source: Parasit Vectors. 2024 Nov 23;17:483. doi: 10.1186/s13071-024-06573-2 (PMC11585139; doi:10.1186/s13071-024-06573-2)
Supplement: Supplementary file 5 — Additional file 5: Fig. S2. CD244 enhances the terminal differentiation and effector phenotype of CD8+ T cells in the spleens of Echinococcus multilocularis-infected mice after 24 weeks of infection. [file 13071_2024_6573_MOESM5_ESM.docx]

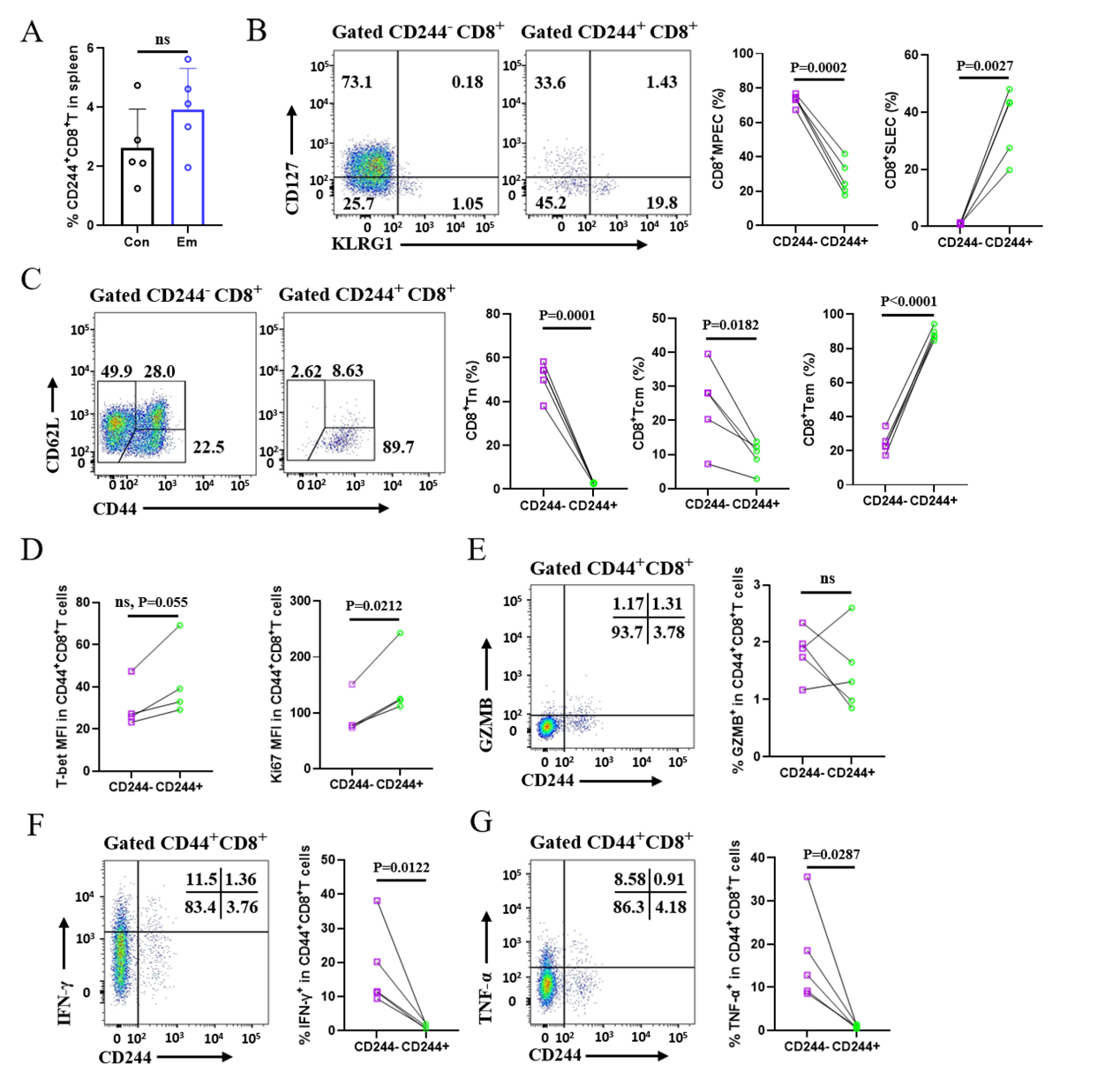


**Fig. S2.** **CD244 enhances the terminal differentiation and effector phenotype of CD8^+^ T cells in the spleens of *E. multilocularis*-infected mice after 24 weeks of infection.** (A) Changes in CD244 expression on CD8^+^ T cells in the spleens of mice infected with *E. multilocularis*. (B) Representative flow cytometry plots (left), and the frequency (right) of MPECs and SLECs in splenic CD244^-^ or CD244^+^ CD8^+^ T cells from mice after 24 weeks of infection (5 mice per group). (C) Representative flow cytometry plots (left), and the frequency (right) of CD8^+^ Tn, CD8^+^ Tcm, and CD8^+^ Tem by CD244^-^ or CD244^+^ CD8^+^ T cells in the spleens of mice after 24 weeks of infection (5 mice per group). (D) MFI of T-bet and Ki67 expression by activated CD244^-^ or CD244^+^CD8^+^ T cells in the spleens of mice after 24 weeks of infection (4 mice per group). (E-G) Representative flow cytometry plots (left), the frequency (right) of GZMB, IFN-γ and TNF-α production by activated CD8^+^ T cells in spleens from mice after 24 weeks of infection (5 mice per group). Data are one representative of two independent experiments. Con, control; Em, *E. multilocularis*. All data are presented as mean ± SD. Data were analyzed using two independent samples t-test or paired Student’s t tests. ns, P > 0.05.
