# Supplementary material for: Upregulation of CD244 promotes CD8+ T cell exhaustion in patients with alveolar echinococcosis and a murine model
Source: Parasit Vectors. 2024 Nov 23;17:483. doi: 10.1186/s13071-024-06573-2 (PMC11585139; doi:10.1186/s13071-024-06573-2)
Supplement: Supplementary file 6 — Additional file 6: Fig. S3. FCM assessed changes in the secretion capacities of GZMB, IFN-γ, TNF-α and IL-10 by liver NK cells and CD4+ T cells from control, WT and CD244-KO mice following 48 h of in vitro Emp stimulation. [file 13071_2024_6573_MOESM6_ESM.docx]

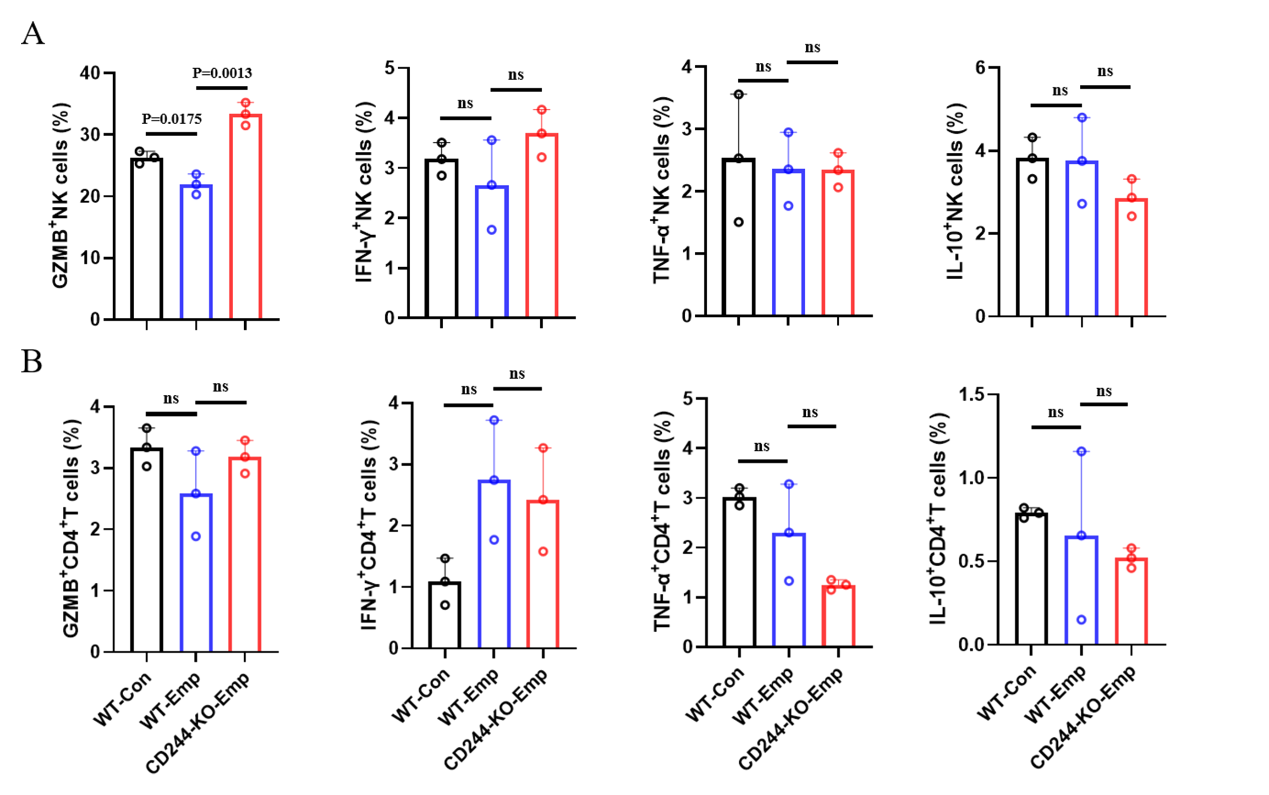


**Fig. S3.** **FCM assessed changes in the secretion capacities of GZMB, IFN-γ, IL-10, and TNF-α by liver NK cells and CD4^+^ T cells from control, WT and CD244-KO mice following 48 hours of in vitro Emp stimulation.** Data are one representative of two independent experiments. KO, knockout; WT, wild type; Emp, *E. multilocularis* protoscoleces. All data are presented as mean ± SD. Data were analyzed using two independent samples t-test. ns, P > 0.05.
