# Supplementary material for: Upregulation of CD244 promotes CD8+ T cell exhaustion in patients with alveolar echinococcosis and a murine model
Source: Parasit Vectors. 2024 Nov 23;17:483. doi: 10.1186/s13071-024-06573-2 (PMC11585139; doi:10.1186/s13071-024-06573-2)
Supplement: Supplementary file 7 — Additional file 7: Fig. S4. CD244 deficiency prevents terminal differentiation and functional exhaustion of CD8+ T cells in the spleens of Echinococcus multilocularis-infected mice at 19 weeks post-infection. [file 13071_2024_6573_MOESM7_ESM.docx]

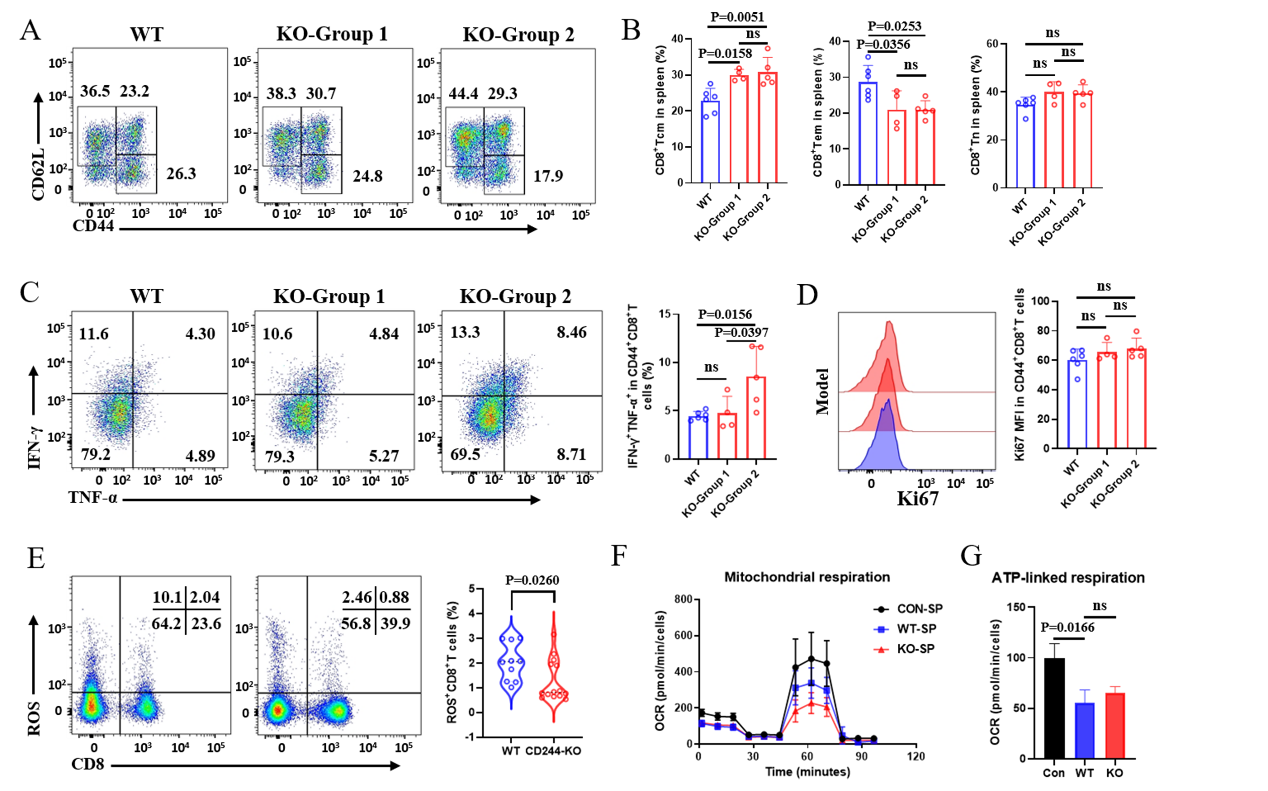


**Fig. S4. CD244 deficiency prevents terminal differentiation and functional exhaustion of CD8^+^ T cells in the spleens of *E. multilocularis*-infected mice at 19 weeks post-infection.** (A, B) Representative flow cytometry plots (left) and frequencies (right) of differentiation phenotypes of CD8^+^ T cells in the spleens of mice at 19 weeks post-infection with *E. multilocularis* (4-6 mice per group). (C) Representative flow cytometry plots and percentages of IFN-γ and TNF-α production by activated CD8^+^ T cells in the spleens of mice after 19 weeks of infection (4–6 mice per group). (D) MFI of Ki67 expression by activated CD8^+^ T cells in the spleens of mice after 19 weeks of infection (4-6 mice per group). (E) Flow cytometry assessment of splenic ROS^+^CD8^+^ T cells from CD244-KO and WT mice after 19 weeks of *E. multilocularis* infection (10-14 mice per group) (F) OCR of spleen CD8^+^ T cells from *E. multilocularis*-infected mice was measured following the same treatment as on liver CD8^+^ T cells. (G) Statistical analysis of mitochondrial ATP-linked respiration in spleen CD8^+^ T cells from mice infected with *E. multilocularis*. Data are one representative of two independent experiments. KO, knockout; WT, wild type. Data were analyzed using one-way ANOVA test or Kruskal-Wallis test. ns, P > 0.05.
