# Supplementary material for: Upregulation of CD244 promotes CD8+ T cell exhaustion in patients with alveolar echinococcosis and a murine model
Source: Parasit Vectors. 2024 Nov 23;17:483. doi: 10.1186/s13071-024-06573-2 (PMC11585139; doi:10.1186/s13071-024-06573-2)
Supplement: Supplementary file 8 — Additional file 8: Fig. S5. CD244 deficiency does not significantly affect the differentiation of donor CD8+ T cells following adoptive transfer into Echinococcus multilocularis-infected CD8-KO mice. [file 13071_2024_6573_MOESM8_ESM.docx]

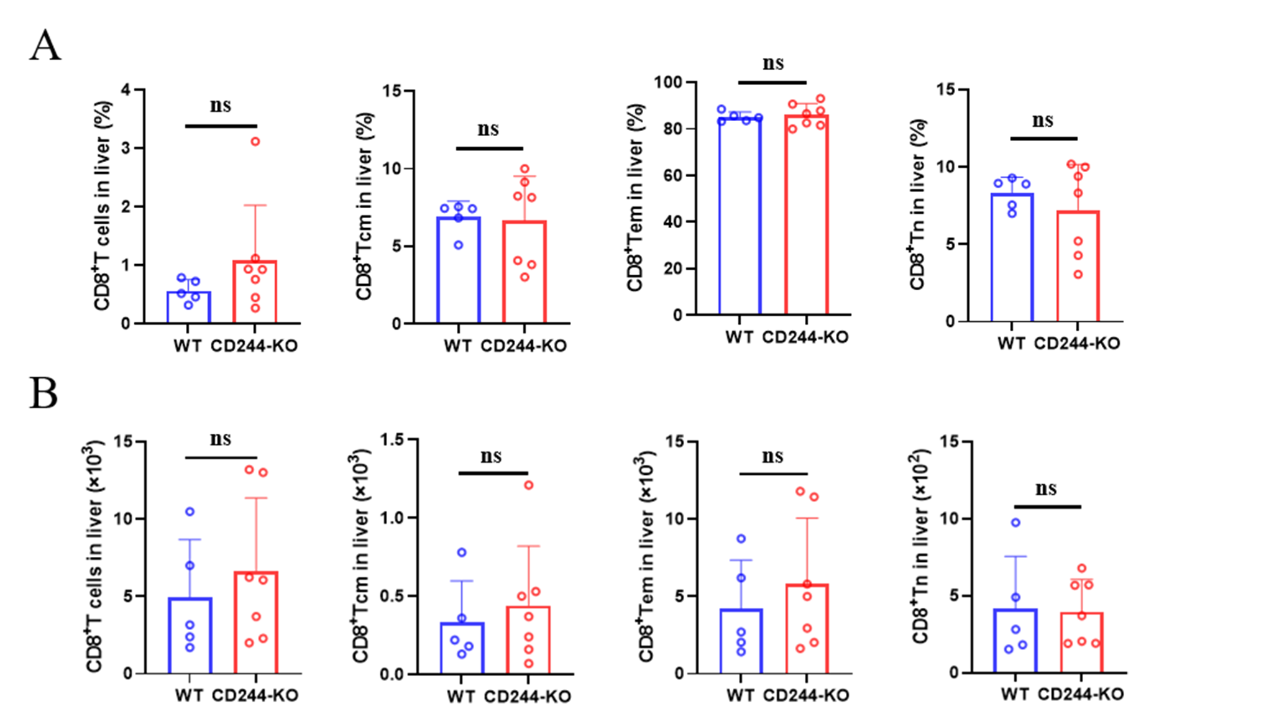


**Fig. S5.** **CD244 deficiency does not significantly affect the differentiation of donor CD8^+^ T cells following adoptive transfer into *E. multilocularis*-infected CD8-KO mice.** (A, B) The percentages and absolute numbers of transferred CD8^+^ T cells, CD8^+^ Tcm, CD8^+^Tem and CD8^+^ Tn in the livers of *E. multilocularis*-infected CD8-KO mice who received adoptive transfers. KO, knockout; WT, wild type. Data were analyzed using two independent samples t-test or Mann Whitney test. ns, P > 0.05.
