# Supplementary material for: Upregulation of CD244 promotes CD8+ T cell exhaustion in patients with alveolar echinococcosis and a murine model
Source: Parasit Vectors. 2024 Nov 23;17:483. doi: 10.1186/s13071-024-06573-2 (PMC11585139; doi:10.1186/s13071-024-06573-2)
Supplement: Supplementary file 9 — Additional file 9: Fig. S6. Flow cytometric gating strategies. [file 13071_2024_6573_MOESM9_ESM.docx]

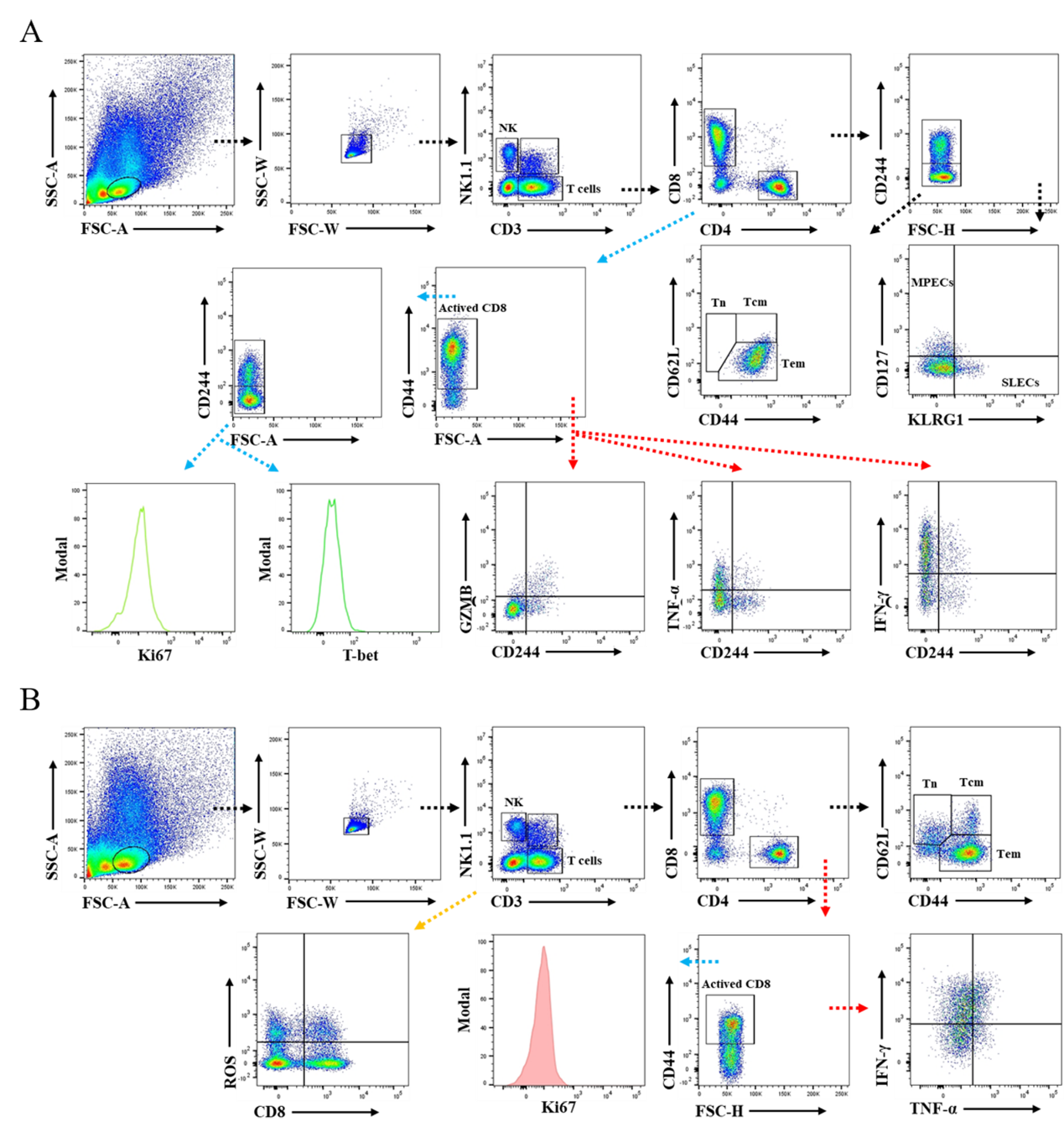


**Fig. S6.** **Flow cytometric gating strategies.** (A) Flow cytometric gating strategy used to identify the differentiation phenotype and functional changes of CD244^-^ or CD244^+^CD8^+^T cells in hepatic and splenic lymphocytes from WT mice with *E. multilocularis* infection. (B) Flow cytometric gating strategy used to identify the differentiation phenotypes, effector functions, and changes in ROS levels of CD8^+^ T cells in hepatic and splenic lymphocytes from WT or CD244-KO mice with *E. multilocularis* infection. *E. multilocularis*: *Echinococcus multilocularis*; WT: wild type; KO: knockout; SLECs: short-lived effector cells; MPECs: memory precursor effector cells; Tem: effector memory T cells; Tcm: central memory T cells.
